# Missed Opportunities for Early Access to Care of HIV-Infected Infants in Burkina Faso

**DOI:** 10.1371/journal.pone.0111240

**Published:** 2014-10-31

**Authors:** Malik Coulibaly, Nicolas Meda, Caroline Yonaba, Sylvie Ouedraogo, Malika Congo, Mamoudou Barry, Elisabeth Thio, Issa Siribié, Fla Koueta, Diarra Ye, Ludovic Kam, Stéphane Blanche, Phillipe Van De Perre, Valériane Leroy, MONOD Study Group ANRS 12206

**Affiliations:** 1 Projet MONOD ANRS 12206, Centre de Recherche Internationale pour la Santé, Site ANRS Burkina, Université de Ouagadougou, Ouagadougou, Burkina Faso; 2 Centre Muraz, Bobo Dioulasso, Burkina Faso; 3 Service de Pédiatrie, CHU Yalgado Ouédraogo, Ouagadougou, Burkina Faso; 4 Service de Pédiatrie Médicale, CHU Charles de Gaulle, Ouagadougou, Burkina Faso; 5 Laboratoire de Bactériologie - Virologie CHU Yalgado Ouédraogo, Ouagadougou, Burkina Faso; 6 Service de laboratoire, CHU Charles de Gaulle, Ouagadougou, Burkina Faso; 7 Groupe hospitalier Necker- Enfants malades, Paris, France; 8 Inserm U1058, Université Montpellier 1, Montpellier, France; 9 Inserm, U897, Institut de Santé Publique, Epidémiologie et Développement (ISPED), Université de Bordeaux, Bordeaux, France; Alberta Provincial Laboratory for Public Health/University of Alberta, Canada

## Abstract

**Objective:**

The World Health Organization (WHO) has recommended a universal antiretroviral therapy (ART) for all HIV-infected children before the age of two since 2010, but this implies an early identification of these infants. We described the Prevention of Mother-to-Child HIV Transmission (PMTCT) cascade, the staffing and the quality of infrastructures in pediatric HIV care facilities, in Ouagadougou, Burkina Faso.

**Methods:**

We conducted a cross-sectional survey in 2011 in all health care facilities involved in PMTCT and pediatric HIV care in Ouagadougou. We assessed them according to their coverage in pediatric HIV care and WHO standards, through a desk review of medical registers and a semi-structured questionnaire administered to health-care workers (HCW).

**Results:**

In 2011, there was no offer of care in primary health care facilities for HIV-infected children in Ouagadougou. Six district hospitals and two university hospitals provided pediatric HIV care. Among the 67 592 pregnant women attending antenatal clinics in 2011, 85.9% were tested for HIV. The prevalence of HIV was 1.8% (95% Confidence Interval: 1.7%–1.9%). Among the 1 064 HIV-infected pregnant women attending antenatal clinics, 41.4% received a mother-to-child HIV transmission prevention intervention. Among the HIV-exposed infants, 313 (29.4%) had an early infant HIV test, and 306 (97.8%) of these infants tested received their result within a four-month period. Among the 40 children initially tested HIV-infected, 33 (82.5%) were referred to a health care facility, 3 (9.0%) were false positive, and 27 (90.0%) were initiated on ART. Although health care facilities were adequately supplied with HIV drugs, they were hindered by operational challenges such as shortage of infrastructures, laboratory reagents, and trained HCW.

**Conclusions:**

The PMTCT cascade revealed bottle necks in PMTCT intervention and HIV early infant diagnosis. The staffing in HIV care and quality of health care infrastructures were also insufficient in 2011 in Ouagadougou.

## Introduction

Despite the efficacy of Prevention of Mother-To-Child- HIV Transmission (PMTCT), Human Immunodeficiency Virus (HIV) pediatric infection still occurs in Africa because of the lack of operational access to this intervention. Without any intervention, mortality of HIV infected children can reach up to 35% before the first birthday and up to 52% before the second birthday [1], [2], and the untreated survivors would need substantial care [3]. However early antiretroviral treatment routinely started before 12 weeks of age significantly increases infant survival by 76%, reduces morbidity and enhances immunological benefits [4], [5], [6], [7]. The 2010 World Health Organization (WHO) revised guidelines recommend early antiretroviral treatment in all HIV infected children less than two years of age, regardless of their immune status [8]. These guidelines also recommend a routine Early Infant Diagnosis (EID) from six-weeks of age of all HIV-exposed children. EID requires sophisticated technology before 18 months of age because of the persistence of maternal antibodies in infants [9]. In addition, the uptake at each step in the EID cascade highlights that even with the highest reported level of uptake, nearly half of HIV-infected infants may not successfully complete the cumulative cascade [10]. In sub-Saharan Africa, HIV-exposed infants continue to suffer from insufficient access to EID and antiretroviral therapy. In 2010, a survey was conducted in Burkina Faso, Ghana and Côte d'Ivoire, to identify the major challenges regarding HIV prophylaxis for children in West Africa [11]. The results of this survey indicated that only a small proportion of HIV-exposed newborns received antiretroviral prophylaxis. Scaling-up management of early pediatric HIV infection remains challenging in West African countries in 2011. But there is a need to increase the PMTCT coverage and to trace the children born in the setting of the PMTCT programs [12]. It is crucial to identify the barriers at the national level. Burkina Faso is a West-African developing country where HIV prevalence was about 1.0% in 2010 [13]. HIV EID in children born to HIV-infected mothers is organized in cascade from the district health care facilities, towards district hospital laboratories, to the university hospital laboratories. There are few data on the full PMTCT cascade coverage and postnatal services in regard to infants born to HIV-infected mothers. Problems are related to resource management, and lack of assessment of sites.

We described the access to pediatric HIV diagnosis and care in Ouagadougou, the capital of Burkina Faso. We also assessed the health care facilities regarding the conformance of staff and infrastructures with WHO standards for the care of HIV infected infants in Ouagadougou in 2010–2011.

## Methods

### Access to HIV care for infants in Burkina Faso

Burkina Faso is administratively divided into 13 regions, 45 provinces and 351 rural and urban municipalities. In 2011, the public health system was organized around four types of hospitals: district, confessional, regional, and university hospitals. Besides the public health facilities, Burkina Faso had also a large number of private health care facilities and traditional healers [14].

The “big” Ouagadougou equated the Center region with a population of 2 136 582 inhabitants in 2011, of whom 39.7% were children less than 15 years of age [14]. The Center region was the most populous and urbanized of the 13 administrative regions of Burkina Faso, with a land area of 2 869 square kilometers [15]. We identified in this region two university hospitals, five district hospitals, one confessional hospital, eight hospitals without surgical units, and 81 primary health care facilities [16]. According to the 2010 health and demographic survey, the HIV prevalence in Ouagadougou was estimated at 2.1% (95% CI: 1.5–2.7) in 2010 [13].

PMTCT services are integrated in the national health system. All pregnant women who come for antenatal care in a health care facility are expected to be counseled for HIV testing. In case of consent, HIV screening is performed on-site using rapid HIV antibody tests, with a simultaneous HIV result delivery. The pregnant women who attend antenatal consultation with a documented positive result are also tested for the sake control, unless that they are already treated with antiretroviral drugs. In any case, there were included in the PMTCT cascade.

In Burkina Faso, the option A of PMTCT was still recommended in 2011 and HIV-infected pregnant women were eligible to antiretroviral treatment on the basis of CD4 count. Pregnant women with more than 350 cells/mm^3^ CD4 count were prescribed zidovudine at 28 weeks gestation in antepartum. In intrapartum, at onset of labor, a single dose of nevirapine and the first dose of zidovudine/lamivudine were given. In postpartum, a daily zidovudine/lamivudine was given for seven days. Whenever the pregnant women's CD4 count was inferior or equal to 350 cells/mm^3^ a triple antiretroviral therapy was started as soon as diagnosed, and continued for life. Infants received a daily nevirapine dose from birth up to one week after the complete cessation of breastfeeding. When mothers were not breastfeeding or were on antiretroviral drugs, infants were given *a* daily nevirapine dose up to six weeks of age. Infant breastfeeding was the most recommended feeding option.

After delivery, HIV-infected mothers and their children were advised to go to the nearest health care facility for a postnatal visit and an EID preformed since six weeks of age. This EID was based on a first deoxyribonucleic acid polymerase chain reaction (DNA PCR) test on a Dried Blood Spot (DBS) and was scheduled once a month. The DBS were sent for processing by the corresponding district hospital to one of the three laboratories in Ouagadougou region (the university hospital Yalgado Ouédraogo, the university hospital Charles de Gaulle, and the Saint Camille hospital). All HIV tests results were sent back to the health care facilities via the corresponding district hospitals. In case of a first positive DBS result, a second DNA PCR test is performed on a blood sample to confirm HIV infection [17].

### Context and study design

The study was conducted in the implementation phase of the MONOD ANRS 12206 clinical trial (ClinicalTrial.gov registry n°NCT01127204), which was approved by the national ethics committee and the Burkina Faso Ministry of Health. The study was conducted in the capital of the country (Ouagadougou). Health professionals who were interviewed and parents of children who were enrolled for treatment provided a clear written consent. The ethics committee approved the consent procedure. The informed consent was waived for the use of aggregate register data.

The ANRS 12206 MONOD trial is a randomized controlled trial whose aim is to assess a simplified once daily antiretroviral treatment in virologically suppressed HIV infected children initially treated with a triple therapy containing lopinavir/ritonavir before the age of two (Appendix S1).

We undertook a cross-sectional survey from January 2011 to January 2012, to evaluate the performance of PMTCT cascade, and the conformance of infrastructures and staff in health care facilities with pediatric HIV services in Ouagadougou.

### Study site and population

We included all the health care facilities providing PMTCT services, infant HIV diagnosis and antiretroviral treatments in Ouagadougou. We first used their 2011 aggregate data to document the PMTCT cascade. Then, in each health care facility, we interviewed all the heads of the various services: health districts, health care facilities, PMTCT services, laboratories, pharmacies, pediatric services, statistics and epidemiology surveillance division, and human resource services.

### Data collection

We used 2010 and 2011 Ministry of Health statistical yearbook, as the reference figures [14], [16]. We designed a semi-structured questionnaire with three sections according to the staff targeted: pediatric, laboratory and pharmacy services. Two medical epidemiologists, one midwife and one sociologist carried out the desk review and interviewed the selected health professionals. The questionnaire reviewed variables related to PMTCT statistics (cascade of HIV care from antenatal services to EID of HIV-exposed children at six weeks of age and antiretroviral treatment care for HIV-infected children), infrastructures, laboratory reagents, essential drug management and health professionals staffing (doctors, nurses, pharmacists, and laboratory technicians). The staff interviews helped to check registers and identify difficulties faced in providing early HIV infant diagnosis and treatment, and possible solutions.

In 2011, there were 103 health care facilities providing PMTCT services in Ouagadougou and we collected data from all of them. In each health care facility, there was a statistics manager who was in charge of collecting data monthly in a register provided by the Ministry of Health. Pregnant women received for antenatal consultation were recorded in a register that was later used by the statistics manager. A report was then sent to the district head of statistics and epidemiology surveillance division, who compiled the different health care facility reports with Excel software. In our study, we monthly recorded data in term of aggregate number of the different variables related to the PMTCT cascade, from the health care facility registers as well as the district registers, for comparison. In case of discrepancies, we monitored the data recording process to correct the errors. Finally, we were able to document individual data for the HIV-infected children diagnosed and transferred to HIV pediatric care for antiretroviral treatment in the MONOD trial.

For drug management, we checked the registers where the drug management was recorded to determine the availability of drugs and stock-outs. We checked the availability of antiretroviral drugs needed for the national guideline treatment: zidovudine or stavudine or abacavir, lamivudine or emtricitabine, nevirapine or efavirenz, and lopinavir/ritonavir. For opportunistic infection treatment and prophylaxis, we checked the availability of the following drugs: cotrimoxazole, nistatine, miconazole, amphotericine B, ciprofloxacine, ceftriaxone, acyclovir, and anti-tuberculosis drugs.

Finally, we checked the laboratory reagent management and availability with the responsible of laboratories in the corresponding registers.

To document the PMTCT cascade, we used the Ministry of Health method to estimate the expected number of pregnancies, based on the expected number of births multiplied by 1.10 [14]. The expected number of births is equal to the number of women of childbearing age multiplied by the corrected fertility rate of the Center region, equal to 0.1247. The logic of multiplying the expected number of births by 1.10 to obtain the number of expected pregnancies comes from a study of Sedgh et al. who found that 10% of pregnancies end in abortions in Western Africa [18].

### Essential infrastructure requirement for health care centers

In 2008, WHO published an operational manual for HIV high prevalence resource constraint settings, to assess health care facilities serving HIV infected people [19].

The WHO defines health care facility's space, design, power supply, water, hygiene and sanitation, and equipment requirements to be able to deliver quality HIV prevention, care and treatment services. We assessed health care facilities using the following WHO criteria: space, privacy and confidentiality, infection control (tuberculosis infection and HIV infection), safe water supply and hygiene (sanitation, hand washing and other hygiene practices, waste management, latrine/toilet, and cleaning), communications, power, and fire safety. The standards require using color-coded waste containers and fire extinguishers. Space requirement is at least 9 m^2^ for consultation room, 2.25 m^2^ for counseling room, 9 m^2^ for laboratory specimen analysis room, and 9 m^2^ for pharmacy room [19].

We checked the space available in pediatric consultation ward, laboratory, and pharmacy rooms. This criteria was classified as conform if the available space was superior or equal to that required by the WHO standards. We also checked other qualitative criteria such as the availability of power supply, infection control and the respect of privacy and confidentiality by health professionals. The conformance was good if all the criteria were met. For laboratory tests, we assessed the capacity for performing the required tests in hospitals, without neither shortages of laboratory reagents nor failure of medical devices.

Finally, the conformance was good if antiretroviral drugs and drugs for opportunistic infection treatment and prophylaxis were available to treat HIV infected children with a regimen recommended by the national guidelines.

### Statistical analysis

The prevalence of HIV infected pregnant women was calculated by dividing the number of HIV infected pregnant women by the total number of pregnant women screened for HIV infection. The 95% confidence intervals were determined according to the following formula: 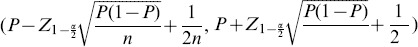 [20] where p =  prevalence, n =  sample size. We described the coverage of pediatric HIV services and the flow from HIV-exposed children to access to ART, of HIV-infected children. The cascade of care was compiled on Microsoft Excel software using proportions. All the proportions of the PMTCT cascade were calculated by dividing the total number of favorable cases by the number of eligible cases with their 95% confidence intervals according to the formula previously mentioned.

## Results

From the 103 health care facilities providing PMTCT services in Ouagadougou in 2011, 127 health professionals were interviewed: 7 (5.5%) pediatricians, 5 (3.9%) general practitioners, 10 (7.9%) pharmacists, 5 (3.9%) nurse-epidemiologists, 75 (59.1%) nurses, 7 (5.5%) midwives, 9 (7.1%) pharmacist assistants, 5 (3.9%) laboratory technicians, 2 (1.6%) biologists, and 2 (1.6%) human resource managers.

### Staffing in pediatric HIV health services

In 2010, there was no HIV treatment for HIV-infected children in primary health care facilities in Ouagadougou. All pediatric HIV care was provided by the six district hospitals, and two university hospitals. In these hospitals, a total of 225 health professionals were directly involved in pediatric HIV infection care, and among them 40% worked in the two university hospitals (Table 1). Overall, 10.7% were pediatricians, 4.4% general practitioners, 19.1% nurses, 5.8% counselors, 8.9% pharmacists, and 29.8% laboratory technicians. Six of the eight hospitals had at least one pediatrician.

**Table 1:** Staff involved in HIV pediatric care per health district or university hospitals, and qualification, in Ouagadougou, in 2010.

	Pediatricians	General	Pharmacists	Laboratory	Psychologist/	Nurses	Others	Total
		practitioners		technicians	counselors			
District hospitals	8	9	6	38	6	30	36	133
Mean per hospital	1.6	1.8	1.2	7.6	1.2	6	7.2	26.6
University hospital	16	1	14	29	7	13	12	92
Mean per hospital	8	0.5	7	14.5	3.5	6.5	6	46

Others: pharmacy assistants and laboratory assistants.

In 2010, the total population of children less than 15 years old in the whole region was estimated at 811 115 [16]. With an HIV prevalence of 0.26% in children less than 15 years old [11], we estimated the number of HIV infected children less than 15 to be about 2 109 (811 115×0.26%) in Ouagadougou. With 24 pediatricians in this area (10.7% of the overall staff dedicated to pediatric HIV care), one pediatrician was responsible for 33 797 (811 115/24) children less than 15 years age of whom 88 (2 109/24) were HIV infected.

When evaluating the conformance of health staff requirements of the WHO standards in the health district hospitals [19], the staff is overall insufficient: general practitioners are less than 1/10 000, and pharmacists are less than 1/20 000 in all the five health districts of Ouagadougou. We had more than 1/4000 nurses in four health districts. Overall, both physician and pharmacist staff were scarce.

### PMTCT cascade

In 2011, out of the 76 935 expected pregnancies in the Center region, 67 592 attended at least one antenatal consultation (87.8%). Among the pregnant women attending antenatal consultation, 58 036 accepted to be HIV-tested (85.9%) and the HIV prevalence was 1.8% (95% CI: 1.7%–1.9%). Furthermore, 441 out of the 1 064 HIV-infected pregnant women (41.4%) benefitted from a PMTCT intervention (option A). Then, only 313 (29.4%) HIV-exposed infants (0–18 months) had an HIV virologic test on a DBS, and 306 (97.8%) among these infants tested received their results, usually within a month, but sometimes within a four-month period. Still among the infants tested, 40 children were initially identified as HIV-infected, and 33 (82.5%) out the infants tested, were referred to the MONOD study sites before the age of two for an HIV test confirmation using a deoxyribonucleic acid polymerase chain reaction (DNA PCR). With three children identified as false positive (9%) and 30 (91%) confirmed to be HIV-infected, the HIV prevalence was estimated to be 9.6% (95% CI: 6.3%–12.9%). Finally, 27 children (90.0%) were enrolled in the MONOD ANRS 12206 trial and treated with a triple lopinavir/ritonavir based therapy (Table 2). Their median age at diagnosis was 13 months [IQR: 7–19].

**Table 2:** The PMTCT cascade until HIV pediatric care in Ouagadougou, 2011.

Designation	Number	Percentage (%)	Confidence
			Interval 95%
Pregnant women expected	76 935	100%	-
Pregnant women attending antenatal	67 592	87.8 (100%)	[87.6–88.0]
consultation			
Pregnant women having been	60 156	89.0	[88.8–89.2]
counseled for HIV testing			
Pregnant women having been HIV-	58 036	85.9 (100%)	[85.6–86.1]
tested		(96.5% of the	
		counseled women)	
HIV-infected pregnant women	1 064	1.8 (100%)	[1.7–1.9]
Pregnant women exposed to PMTCT	441	41.4	[38.4–44.4]
intervention			
HIV-exposed infants having been HIV-	313	29.4 (100%)	[26.6–32.2]
tested using DBS			
DBS tests results returned	306	97.8	[96.0–99.6]
Infants identified as HIV-infected on the	40	12.8	[8.9–16.6]
first DBS test (100%)			
HIV-infected infants referred to pediatric	33	82.5	[71.9–95.5]
care			
Infants confirmed as HIV-infected on the	30	9.6 (100%)	[6.3–12.9]
second test (DNA PCR on blood			
sample)			
HIV-infected infants initiated on	27	90.0	[79.2–100.0]
antiretroviral therapy			

100% is the reference number.

DNA PCR  =  deoxyribonucleic acid polymerase chain reaction.

DBS  =  dried blood spot.

Seven children (17.5%) were not referred for HIV care. One of them died before his laboratory result was released. The six remaining did not come back for their laboratory results and we were not able to contact them because of missing telephone numbers or addresses in the health care facility registers.

Among the six (18.2%) children who were referred to MONOD clinical sites, but were not enrolled in the trial to start an antiretroviral therapy, two died before being able to initiate treatment because of their advanced stage of HIV disease. One was lost to-follow-up after his father refusal to consent for treatment, and three were finally controlled as HIV-negative and not eligible for antiretroviral treatment.

### Infrastructures

The conformance of infrastructures was globally gauged “not conform” because of two criteria: safe waste management and fire safety. Indeed, none of the health care facilities had either segregate color-coded waste containers or fire extinguishers.

### Essential laboratory tests and apparatus

The availability of essential laboratory tests was checked in health care facilities (Table 3). The lack of some of the laboratory tests was associated to either a failure/lack of the corresponding laboratory apparatus or a reagent shortage. The table 4 displays the reasons for the laboratory non conformance. In addition, lack of apparatus maintenance has been underlined in all the health care facilities.

**Table 3:** Conformance of Ouagadougou hospitals according to WHO standards, in 2010.

WHO criteria of conformance	District hospital	University hospital
	N = 6	N = 2
	# conform/N	# conform/N
**Infrastructure**		
Room space >9 m^2^	6/6	2/2
Power (electricity) available	6/6	2/2
Tap water available	6/6	2/2
Tuberculosis infection control (ventilated waiting	6/6	2/2
rooms, cough control, good patient flow)		
HIV infection control (injection safety, appropriate use	6/6	2/2
and disposal of sharps, personal protective equipment		
for staff, post exposure prophylaxis available)		
Waste management (3 color-containers available)	0/6	0/2
Privacy of patient's test protected	6/6	2/2
Communication (land line available)	6/6	2/2
Fire extinguisher available	0/6	0/2
**Conclusion**	**Not conform**	**Not conform**
**Laboratory test available**		
Rapid HIV antibody test	4/6	2/2
DBS	6/6	2/2
CD4 count	1/6	1/2
Hemoglobin determination	5/6	2/2
Serum alanin aminotransferase	4/6	2/2
Serum creatinin & blood urea nitrogen	4/6	2/2
Bilirubin determination	4/6	2/2
Lactic acid	5/6	2/2
Blood sugar	4/6	2/2
Tuberculosis diagnostics	6/6	2/2
Pregnancy test	6/6	2/2
Urine dipstick for sugar and protein	6/6	2/2
**Conclusion**	**Not conform**	**Not conform**
**Drugs available**		
Antiretroviral drugs	6/6	2/2
Opportunistic infection drugs	6/6	2/2
**Conclusion**	**Conform**	**Conform**
**Staff**		
One general practitioner for 10 000	0/6	Not applicable*
One pharmacist for 20 000	0/6	Not applicable*
One nurse for 4 000	4/6	Not applicable*
**Conclusion**	**Not conform**	**Not applicable**

*The university hospitals are located in the center region, but patients are referred from the whole country.

**Table 4:** Reasons for Ouagadougou laboratory non conformance in 2010.

	District hospitals N = 6		University hospitals N = 2	
Laboratory tests non available	Lack or apparatus failure	Reagent shortage	Lack or apparatus failure	Reagent shortage
Rapid HIV antibody test	Non applicable	2	Non applicable	0
CD4 count	5	5	1	0
Hemoglobin	1	0	0	0
determination				
Serum alanin	1	1	0	0
aminotransferase				
Serum creatinin & blood	1	1	0	0
urea nitrogen				
Bilirubin determination	1	1	0	0
Lactic acid	1	0	0	0
Blood sugar	1	1	0	0

### Essential drugs

The availability of essential antiretroviral drugs was quite good in 2010, and the conformance was judged to be good in spite of few shortages which did not affect the treatment of HIV-infected patient according to national guidelines. A shortage of seven days was observed for lopinavir/ritonavir, lamivudine and abacavir in Charles de Gaulle university hospital in 2010. In addition, Bogodogo district hospital noticed a shortage of 30 days for the combination lamivudine + nevirapine + stavudine (triomune junior). The drugs for opportunistic infections were available and conform to WHO standards, but they were not free of charge for HIV-infected patients in 2010, except anti-tuberculosis drugs and cotrimoxazole.

## Discussion

This cross-sectional survey assessed for the first time the staff, the infrastructures and the PMTCT cascade from prenatal PMTCT up to pediatric HIV care, in all health care facilities providing pediatric HIV care services in Ouagadougou. We documented that only 40% of HIV-infected women received a PMTCT intervention and less than a third of HIV-exposed children were tested during the postnatal period. Moreover, it provides a description of the health care system in this country, useful to understand some of the weaknesses of the system when it comes to the issue of EID in all HIV exposed children, and their access to antiretroviral therapy.

There are several drawbacks in our observations. Firstly, the incompleteness of the data collected may be the source of information bias in this study. Our study method was partially based on desk review, where we checked the statistics in the available registers. Unfortunately, we could not get all the information related to our objectives. For instance, it had not been possible to routinely determine the duration of laboratory reagent shortage. Secondly, we were not able to really link one-to-one the PMTCT with the postnatal data, and we assumed that each HIV-pregnant woman was supposed to give one alive pregnancy outcome, without taking into account multiple pregnancy outcomes or stillbirth. Furthermore, some of the infants tested in 2011, had their mothers attend their first antenatal consultation in the preceding years, and some of the pregnant women tracked in 2011 will give birth in 2012 as well, resulting in a kind of compensation allowing the PMTCT and EID coverage estimates. Consequently, we feel that these figures were accurate enough to understand the overall patient flow throughout the health care system services. Thirdly, the conformance of health care services was determined with respect to the WHO standards, ideally suitable for district hospitals [19]. These standards might not be suitable when applied to university hospitals, where a higher standard of care is expected. Lastly, in terms of representativeness, our results showed a similar proportion of antenatal consultations among pregnant women in Ouagadougou, compared to the rest of the country (88%) [14]. As a matter of fact, Ouagadougou had a greater number of private health facilities compared to the rest of the country [14], and their statistics were not included in our figures. As a result, the number of antenatal consultations was lower than that was really carried out in Ouagadougou. However, in our study, we considered all pregnant women who were on PMTCT antiretroviral protocol with the hypothesis that their children would be referred to the public system if they were found to be HIV-infected.

Our study helped to identify major challenges facing EID and antiretroviral treatment access for children in Burkina Faso. A survey conducted in Burkina Faso, Ghana and Côte d'Ivoire, from January 2010 to February 2011 had already reported the lack of access to child PMTCT prophylaxis [11]. Our results confirm that in the urban setting of Ouagadougou. The level of missed opportunities was so high that it was difficult to cover sufficiently with PMTCT intervention, the mother-infant couple, estimated at 59%, as well as to offer EID to all HIV-exposed children, reaching 71%. We also conclude that these missed opportunities should be greater at the national level considering the fact that the health care system would be more complex and thus weaker at the rural level in comparison to the urban one.

Some factors could explain these missed opportunities and could be separately addressed. Firstly, the low awareness of HIV prevention and care services in the community could explain the non-attendance of EID services or the rejection of these services by families. In Burkina Faso, only 20.1% of the population attended the secondary school in 2008/2009 [21] and therefore we think that substantial efforts should be developed to make them aware of the benefit of PMTCT services. In addition, men should also be targeted in education, because they are likely to be reluctant to carrying out HIV screening, and they can greatly support their wives in using PMTCT services [22], [23].

Secondly, we highlighted the lack of adequate quantitative and qualitative health care workers (HCW) to cover the needs. At the six-week postnatal visit for instance, due to the fact that nurses were not all trained to perform DBS, the DBS could only be performed one day in a month. Mothers who would like have their infant HIV-tested could only return on this unique day, leading to a high attrition rate because of the inadequate offer of this simple service.

Thirdly, the inaccessibility to EID services is also related to the health system organization, as the current DBS sample circuit and transportation is too complicated and should be simplified. Indeed, the need to go through each district hospital while the final laboratory test is performed in each university laboratory hospital should be considered. It would be more efficient to perform the DBS directly in the health care facilities, to limit the lost to follow-up rate. In the neighboring country of Côte d'Ivoire, we had a similar problem of low coverage rate of EID, favored by the civil war, with only 24% of the HIV exposed children early diagnosed in 2010 [24]. In comparison, a study carried out in 2008, showed that DNA polymerase chain reaction testing in routine was 35.2% for children hospitalized in Malawi, but their age was not specified [25].

Similarly, the low PMTCT intervention coverage is related to problems in the health service organization. When a pregnant woman attends antenatal consultation in a health care facility, she is counseled and screened for HIV with a rapid HIV antibody test. In case of HIV infection, she is referred to the referent district hospital in order to carry out the other tests such as CD4 count, before visiting a doctor who would prescribe an option A antiretroviral treatment, mainly based on nevirapine. Then, she is later sent back in the former health care facility, to pursue her antenatal follow-up. Although not documented, we assume that some pregnant women could not reach the district hospitals, thus increasing the number of lost to follow-up. This could explain why a lot of pregnant women attend antenatal consultation but do not benefit from PMTCT intervention, when they are HIV-infected.

Fourthly, there are also laboratory related challenges, with the need to offer routine services while the HIV-prevalence is still low, leading to frequent laboratory reagent shortages. Thairu et al. also confirmed our results about maintenance and reagent stock management, in their study in Burkina Faso and Zimbabwe [26]. A frequently-cited barrier to expansion of EID programs is the cost of the required laboratory assays.

Thus, substantial sequential barriers explain the low PMTCT and EID complete cascade coverage, and the lack of personnel and infrastructure requirements. A review reported that even with the highest reported levels of uptake, nearly half of HIV-infected infants may not complete the cascade successfully [10].

Additionally, we raise the overall problem of the EID strategy performances. In settings with low HIV prevalence or well performing PMTCT program, vertical transmission rates may be as low as 2% at six weeks and the positive predictive value of a single test will be approximately 50%, meaning that only half of infants who are tested positive are truly infected [27], [28]. For this reason, a confirmatory test is essential, especially in the context of a low HIV prevalence country such as Burkina Faso. Indeed, in our study, the high rate of false positive DBS (9%) highly affects the positive predictive value of the national HIV screening strategy. Acknowledging this, the test confirmation is a priority and laboratories should implement reliable quality control system and constantly work on maintaining high quality standards of EID.

Antiretroviral access for HIV-infected children looked good in our study when compared to the estimates of the Ethiopian study, where only 8.4% of positive babies had access to antiretroviral treatment [29]. But, it is important to point out the contribution of the Monod trial implementation in our results, which set up a network whose aim was to improve the coverage of pediatric antiretroviral therapy beyond the EID.

A shortage of some antiretroviral drugs was observed in two health care facilities for several days, as a result of delays in reporting. In effect, antiretroviral drugs are provided by the Ministry of Health division for HIV/AIDS (Comité Ministériel de Lutte contre le Sida), and they required periodic reports, before delivery. Hence, a delay in providing a report will ultimately end in a delay in drug supply.

Moreover, while all antiretroviral drugs were free of charge in Burkina Faso in 2010, the opportunistic infection drugs are charged to families. It has already been reported that having to pay for HIV treatment and laboratory tests, increases the risk of lost to follow-up [30].

When analyzing the conformance of health centers with respect to the PMTCT cascade, we can point out that the infrastructure requirements are almost met, and that the absence of fire extinguishers and segregate color-codes waste containers, did not affect antenatal consultation rate which is quite good in a developing country setting such as this. However, the non conformance to laboratory test requirements explained why we observed an attrition of the cascade at the number of children tested for HIV infection. The conformance of pharmacies was found to be good and consequently two-third of HIV infected children were treated. The missed opportunity for treatment was related to communication and pregnant women HIV testing circuit problem.

Globally, the causes of non conformance at the district and university hospitals are almost similar because they are public centers (except Saint Camille hospital), run by the Ministry of Health. The causes could be a lack of resources, or a mismanagement of the available resources.

The problem could be alleviated by improving the communication process between the peripheral health services and the national procurement system. The community awareness should also be improved and contextualized to the socio-cultural needs of the region.

Moreover, training in a large scale on DBS practicing and in HIV care among HCW would be useful and promote task shifting activities [31], [32], [33]. Finally, characteristics of the health care facilities could be determinant in improving the pediatric HIV care in Africa as reported in the HEART project [34]: characteristics associated with favorable children enrolment in care are nutritional support, linkages with associations of people living with HIV, access to EID and integration of PMTCT services. Applying the South African strategies to improve antiretroviral treatment in the province of KwaZulu-Natal could be beneficial. In addition to training all the staff in contact with mothers and children, they carried out campaigns aimed at increasing HIV testing during immunization and clinics, routinely testing of HIV in children with tuberculosis and malnutrition, and systematically testing for HIV, all children admitted at hospital [35]. However, in Burkina Faso, as the HIV prevalence is lower than that of South Africa, it would be more efficient to start the systematic HIV diagnosis by screening first the children with rapid HIV antibody tests. Expanding these characteristics to improve pediatric HIV treatment in Burkina Faso, warrants further evaluation for improving the scaling up of pediatric HIV care. Finally, as it was reported in South Africa, it is possible to improve the identification of HIV-infected children and ensure a prompt start on ART when needed with relatively simple measures, limited staffing and budgets [35].

Despite an overall good access to prenatal services in Ouagadougou in 2011, there are still many missed opportunities for both the prevention of mother-to-child transmission and the early access to diagnosis and antiretroviral therapy for HIV-infected children before two years of life. The government should look forward to improving the awareness and education among the population, training health care workers for HIV diagnosis and care, facilitating the access to EID and making health care facilities more attractive to families. In addition, the DBS circuit should be simplified to avoid lost to follow-up. Early access to EID and to antiretroviral therapy will require political willingness and leadership to address these health system barriers in Burkina Faso.

## Supporting Information

Appendix S1 **The ANRS 12206 MONOD Collaboration Study Group.**
(DOC)

## References

[1] Newell ML, Brahmbhatt H, Ghys PD (2004) Child mortality and HIV infection in Africa: a review. AIDS 18 Suppl 2 S27–34. 10.1097/00002030-200406002-00004 15319741

[2] Newell ML, Coovadia H, Cortina-Borja M, Rollins N, Gaillard P, et al (2004) Mortality of infected and uninfected infants born to HIV-infected mothers in Africa: a pooled analysis. Lancet 364: 1236–1243. 15464184 10.1016/S0140-6736(04)17140-7

[3] Desmonde S, Coffie P, Aka E, Amani-Bosse C, Messou E, et al (2011) Severe morbidity and mortality in untreated HIV-infected children in a paediatric care programme in Abidjan, Cote d'Ivoire, 2004–2009. BMC Infect Dis 11: 182. 21699728 10.1186/1471-2334-11-182 PMC3138448

[4] Goetghebuer T, Le Chenadec J, Haelterman E, Galli L, Dollfus C, et al (2012) Short- and long-term immunological and virological outcome in HIV-infected infants according to the age at antiretroviral treatment initiation. Clin Infect Dis 54: 878–881. 22198788 10.1093/cid/cir950

[5] Prendergast AJ, Penazzato M, Cotton M, Musoke P, Mulenga V, et al (2012) Treatment of young children with HIV infection: using evidence to inform policymakers. PLoS Med 9: e1001273. 22910874 10.1371/journal.pmed.1001273 PMC3404108

[6] Prendergast A, Mphatswe W, Tudor-Williams G, Rakgotho M, Pillay V, et al (2008) Early virological suppression with three-class antiretroviral therapy in HIV-infected African infants. AIDS 22: 1333–1343. 18580613 10.1097/QAD.0b013e32830437df

[7] Violari A, Cotton MF, Gibb DM, Babiker AG, Steyn J, et al (2008) Early antiretroviral therapy and mortality among HIV-infected infants. N Engl J Med 359: 2233–2244. 19020325 10.1056/NEJMoa0800971 PMC2950021

[8] WHO (2010) Antiretroviral therapy for HIV infection in infants and children: Towards universal access. Recommendations for a public health approach. 2010 revision. 23741772

[9] Nielsen K, Bryson YJ (2000) Diagnosis of HIV infection in children. Pediatr Clin North Am 47: 39–63. 10697641 10.1016/s0031-3955(05)70194-2

[10] Ciaranello AL, Park JE, Ramirez-Avila L, Freedberg KA, Walensky RP, et al (2011) Early infant HIV-1 diagnosis programs in resource-limited settings: opportunities for improved outcomes and more cost-effective interventions. BMC Med 9: 59. 21599888 10.1186/1741-7015-9-59 PMC3129310

[11] Tchidjou HK, Maria Martino A, Goli LP, Diop Ly M, Zekeng L, et al (2012) Paediatric HIV infection in Western Africa: the long way to the standard of care. J Trop Pediatr 58: 451–456. 22529318 10.1093/tropej/fms015

[12] Ndondoki C, Brou H, Timite-Konan M, Oga M, Amani-Bosse C, et al (2013) Universal HIV screening at postnatal points of care: which public health approach for early infant diagnosis in Cote d'Ivoire? PLoS One 8: e67996. 23990870 10.1371/journal.pone.0067996 PMC3749176

[13] Ministère de l'Economie et des Finances Burkina Faso (2011) Enquête Démographique et de Santé et à indicateurs Multiples (EDSBF-MICS IV) 2010. Ouagadougou, Burkina Faso. 50 p. Available: www.measuredhs.com/pubs/pdf/PR9/PR9.pdf. Accessed 2012 September 12.

[14] Ministère de la Santé Burkina Faso (2012) Annuaire statistique 2011. Ouagadougou, Burkina Faso. 244 p. Available: http://www.sante.gov.bf/phocadownload/Annuaire_statistique_2011.pdf. Accessed 2013 May 27.

[15] Institut National de la Statistique et de la Démographie Burkina Faso (2011) La région du centre en chiffres. Ouagadougou, Burkina Faso. 7 p. Available: http://www.insd.bf/n/contenu/statistiques_regions/regions_en_chiffres_en_2011/reg_chif_c_2011.pdf. Accessed 2014 August 4.

[16] Ministère de la Santé Burkina Faso (2011) Annaire statistique 2010. Ouagadougou, Burkina Faso. 204p. Availaible: http://www.sante.gov.bf/phocadownload/Publications_statistiques/Annuaire/annuaire_statistique_sante_2010.pdf. Accessed: 2014 October 5.

[17] Ministère de la Santé Comité Ministériel de Lutte contre le SIDA Burkina Faso (2008) Normes et protocoles de prise en charge médicale des personnes vivant avec le VIH au Burkina Faso. Ouagadougou.

[18] Sedgh G, Henshaw S, Singh S, Ahman E, Shah IH (2007) Induced abortion: estimated rates and trends worldwide. Lancet 370: 1338–1345. 17933648 10.1016/S0140-6736(07)61575-X

[19] WHO (2008) Operations Manual for Delivery of HIV Prevention, Care and Treatment at Primary Health Centres in High-Prevalence, Resource-Constrained Settings. Geneva, Switzerland. 392 p. 26290930

[20] Forthofer RN, Lee ES, Hernandez M (2007) Biostatistics: A guide to Design, Analysis, and Discovery: Elsevier. 502 p.

[21] Institut National de la Statistique et de la Démographie Burkina Faso (2013) Tableau 05.34: Evolution du taux brut de scolarisation de l'ensemble du secondaire (en %). Ouagadougou, Burkina Faso. Available: http://www.insd.bf/n/contenu/tableaux/T0534.htm. Accessed 2013 May 27.

[22] Desgrees-Du-Lou A, Brou H, Djohan G, Becquet R, Ekouevi DK, et al (2009) Beneficial effects of offering prenatal HIV counselling and testing on developing a HIV preventive attitude among couples. Abidjan, 2002–2005. AIDS Behav 13: 348–355. 17985231 10.1007/s10461-007-9316-6

[23] Brou H, Djohan G, Becquet R, Allou G, Ekouevi DK, et al (2008) Sexual prevention of HIV within the couple after prenatal HIV-testing in West Africa. AIDS Care 20: 413–418. 18449817 10.1080/09540120701867065 PMC2662626

[24] Folquet-Amonissani M, Dainguy M. E, Amani-Bossé C, Elian-Kouakou J, Méa-Assandé V, et al. Early infant diagnosis and access to pediatric HIV care: barriers and challenges in Abidjan, Ivory Coast in 2011; 2012; Washington DC, USA.

[25] Van Rompaey S, Kimfuta J, Kimbondo P, Monn C, Buve A (2011) Operational assessment of access to ART in rural Africa: the example of Kisantu in Democratic Republic of the Congo. AIDS Care 23: 686–693. 21390887 10.1080/09540121.2010.532538

[26] Thairu L, Katzenstein D, Israelski D (2011) Operational challenges in delivering CD4 diagnostics in sub-Saharan Africa. AIDS Care 23: 814–821. 21400312 10.1080/09540121.2010.541416

[27] WHO (2010) WHO recommendations on the diagnosis of HIV infection in infants and children. Geneva, Switzerland. 64 p. 23741779

[28] WHO (2014) March 2014 supplement to the 2013 consolidated guidelines on the use of antiretroviral drugs for treating and preventing HIV infection recommendations for a public health approch. Geneva, Swetzerland. 128 p. 24716260

[29] Nigatu T, Woldegebriel Y (2011) Analysis of the prevention of mother-to-child transmission (PMTCT) service utilization in Ethiopia: 2006–2010. Reprod Health 8: 6. 21496304 10.1186/1742-4755-8-6 PMC3094274

[30] Leroy V, Malateste K, Rabie H, Lumbiganon P, Ayaya S, et al (2013) Outcomes of antiretroviral therapy in children in Asia and Africa: a comparative analysis of the IeDEA pediatric multiregional collaboration. J Acquir Immune Defic Syndr 62: 208–219. 23187940 10.1097/QAI.0b013e31827b70bf PMC3556242

[31] Zachariah R, Ford N, Philips M, Lynch S, Massaquoi M, et al (2009) Task shifting in HIV/AIDS: opportunities, challenges and proposed actions for sub-Saharan Africa. Trans R Soc Trop Med Hyg 103: 549–558. 18992905 10.1016/j.trstmh.2008.09.019

[32] Creek T, Tanuri A, Smith M, Seipone K, Smit M, et al (2008) Early diagnosis of human immunodeficiency virus in infants using polymerase chain reaction on dried blood spots in Botswana's national program for prevention of mother-to-child transmission. Pediatr Infect Dis J 27: 22–26. 18162933 10.1097/INF.0b013e3181469050

[33] Oga MA, Ndondoki C, Brou H, Salmon A, Bosse-Amani C, et al (2011) Attitudes and practices of health care workers toward routine HIV testing of infants in Cote d'Ivoire: the PEDI-TEST ANRS 12165 Project. J Acquir Immune Defic Syndr 57 Suppl 1 S16–21. 21857280 10.1097/QAI.0b013e31821fd487

[34] Adjorlolo-Johnson G, Wahl Uheling A, Ramachandran S, Strasser S, Kouakou J, et al (2013) Scaling up pediatric HIV care and treatment in Africa: clinical site characteristics associated with favorable service utilization. J Acquir Immune Defic Syndr 62: e7–e13. 22955053 10.1097/QAI.0b013e3182706401

[35] Bland RM, Ndirangu J, Newell ML (2013) Maximising opportunities for increased antiretroviral treatment in children in an existing HIV programme in rural South Africa. BMJ 346: f550. 23447354 10.1136/bmj.f550

